# Angiographic assessment of lenticulostriate artery sign to predict clinical outcomes after thrombectomy in patients with stroke

**DOI:** 10.3389/fneur.2025.1644288

**Published:** 2025-08-15

**Authors:** Shen Chen, Lisong Dai, Yiran Zhang, Xiaoer Wei, Bicong Yan, Yuehua Li

**Affiliations:** Institute of Diagnostic and Interventional Radiology, Shanghai Sixth People’s Hospital Affiliated to Shanghai Jiao Tong University School of Medicine, Shanghai, China

**Keywords:** stroke, mechanical thrombectomy, basal ganglia, digital subtracted angiography, lenticulostriate arteries, outcome prediction

## Abstract

**Objective:**

Lenticulostriate artery (LSA) reperfusion is critical for basal ganglia blood supply. Basal ganglia infarction (BGI) inconveniencing patients with large artery occlusion and occluded perforators may influence clinical outcomes. This study aims to investigate the association between LSA recanalization, BGI, and long-term outcome after thrombectomy in the ischemic hemisphere.

**Methods:**

In total, 158 stroke patients who underwent thrombectomy were included in this study. Clinical and imaging variables were retrospectively analyzed. LSA signs were categorized as presence (LSA+) or absence (LSA−) of clear vascular patency in the ischemic hemisphere at on-going and post recanalizations. Logistic regression was used to test the relationship between baseline clinical and imaging variables and BGI (primary outcome). The secondary outcome was 90-day modified Rankin Scale (mRS) >2.

**Results:**

Good functional outcome (mRS ≤2, 41.8%) varied among LSA sign patterns. In the multivariate analysis, LSA sign patterns were significantly associated with both BGI and 90 days mRS >2. The odds ratios of LSA−/− and LSA+/LSA− patterns in BGI and long-term outcome remained significant after adjustment of confounders. Models comprising LSA patterns achieved AUC of 0.74 for BGI and 0.91 for long-term outcome.

**Conclusion:**

LSA signs before and after thrombectomy were significantly associated with BGI and long-term functional outcome. This may be a potential predictor of regional ischemic vulnerability and long-term recovery in patients with stroke.

## 1 Introduction

Ischemic stroke is the leading cause of disability and mortality worldwide (1). Therefore, rapid restoration of blood flow by interventional treatment in the ischemic area is crucial to minimize brain injury and improve clinical outcomes (2, 3). Mechanical thrombectomy has emerged as the standard intervention of choice for the recanalization of large-vessel occlusions (2, 4). However, the success of endovascular treatment is not uniform, and various factors contribute to the variability in long-term clinical outcomes (5, 6).

Accurate prognosis assessment following mechanical thrombectomy is crucial to ensure successful recanalization and guide subsequent management strategies (4). Despite successful recanalization, quite a few patients still experience unfavorable outcomes due to microvascular reperfusion failure and basal ganglia injury (BGI). The modified Rankin Scale (mRS), which evaluates the degree of functional recovery, is widely applied as a standard outcome measure in stroke trials, with the time point of 90 days after therapy being the most commonly adopted approach for assessing long-term disability (2, 4). However, long-term outcome assessment in stroke is inherently delayed, and irreversible complications or disabilities may occur before such evaluations are made. Therefore, the identification of early-phase imaging biomarkers is warranted to enable timely risk stratification and to guide post-treatment management decisions.

Lenticulostriate arteries (LSAs) are small perforating arterioles <1 mm in diameter that arise from the middle cerebral artery (MCA) (7). They supply critical regions involved in motor and sensory functions, such as the basal ganglia and internal capsule (8, 9). Therefore, recanalization of the MCA toward the LSA is crucial for predicting the risk of BGI (10). Angiographic features of LSAs have recently emerged as a feasible strategy for predicting clinical outcomes following MCA recanalization treatment (11, 12). However, most of these studies only considered LSA status before treatment (13–16).

Digital subtraction angiography (DSA) is a real-time neuromonitoring technique used to detect brain vasculature, including small perforators. Studies have suggested several intraoperative angiographic signs observed on DSA during endovascular thrombectomy that are associated with prognosis after discharge (17–19). Several researchers have emphasized the LSA territory, basal ganglia, and its association with the outcome of ischemic stroke (15–20). Nevertheless, the effect of LSA angiographic signs at the pre- and post-treatment stages on clinical outcomes warrants validation.

Therefore, this study aimed to elucidate the impact of LSA reperfusion status on the clinical outcomes of patients with large-vessel occlusions and develop prediction models to guide follow-up management strategies for patients with stroke. We hypothesized that combined assessment of pre- and post-thrombectomy LSA patterns would predict clinical outcomes in patients with large-vessel occlusion.

## 2 Materials and methods

### 2.1 Study enrollment and ethics

**Figure 1:**
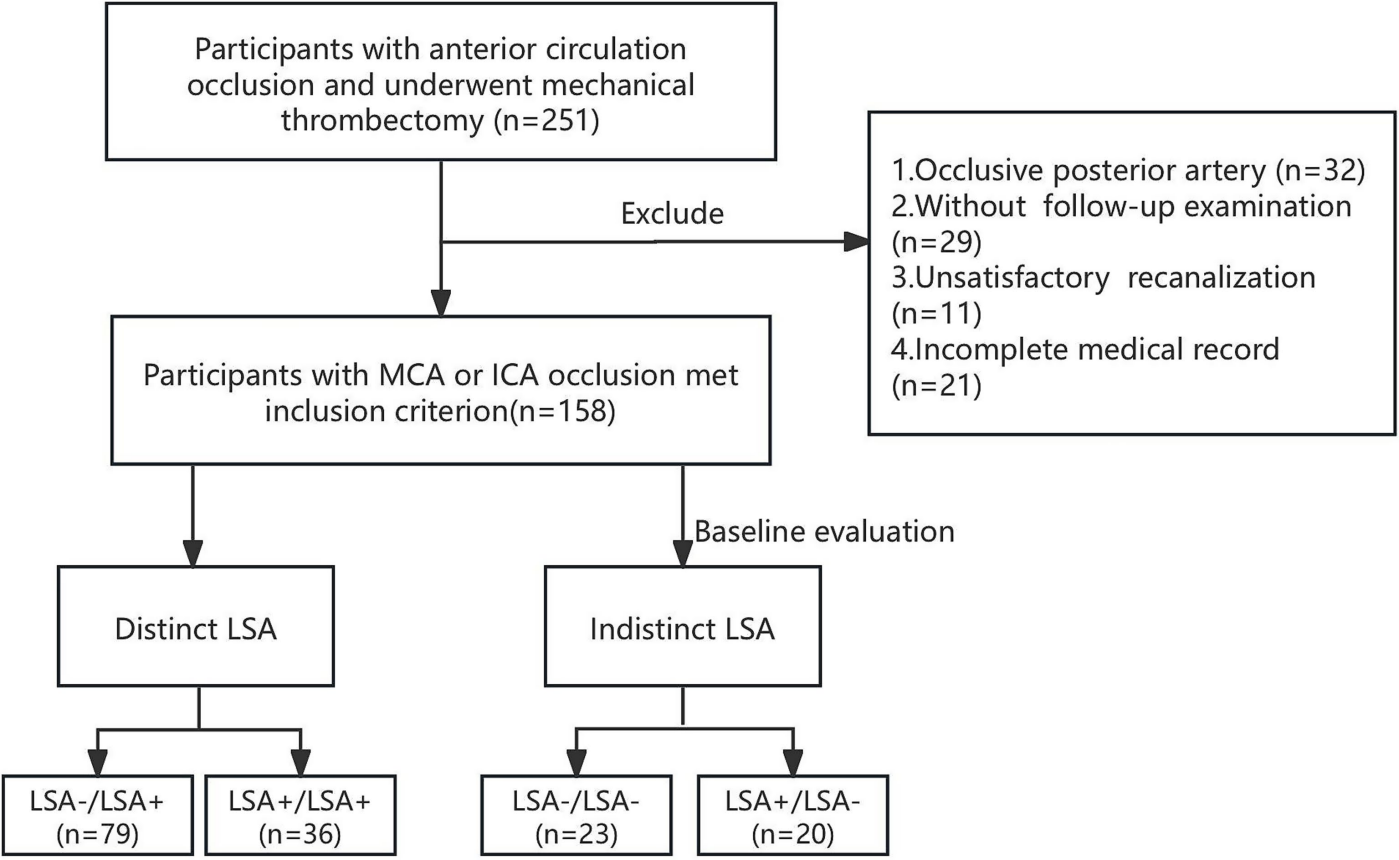
Flowchart of study inclusion and exclusion criteria. MCA, middle cerebral artery. ICA, internal carotid artery; LSA, lenticulostriate artery.

We performed a retrospective cohort study involving patients with anterior circulation stroke who underwent thrombectomy between 2020 and 2022. The patient selection flowchart is shown in Figure 1. The inclusion criteria were as follows: 1. Anterior circulation large-vessel occlusion confirmed by computed tomography (CT) angiography or DSA; 2. Presence of core infarction within 24 h of symptom onset on perfusion CT; 3. Underwent thrombectomy; 4. Achieved satisfactory recanalization, deemed as modified Thrombolysis in Cerebral Infarction (mTICI) score of 2c-3; and 5. Complete follow-up examination with magnetic resonance imaging (MRI) or non-enhanced CT. The exclusion criteria were as follows: 1. Incomplete medical and surgical records; 2. Poor image quality or severe artifacts; and 3. Lost to follow-up. This study was approved by the Shanghai Sixth People’s Hospital Affiliated to Shanghai Jiao Tong University School of Medicine Institutional Ethics Committee [IRB: 2022-KY-130(K)]. Consent for publication was obtained from all participants.

### 2.2 Clinical information

The National Institutes of Health Stroke Scale (NIHSS) on admission and discharge were recorded. The long-term neurological outcomes were assessed using the mRS at discharge and on 90th day. Good and poor clinical outcomes were defined as mRS scores of 0–2 and ≥3, respectively. Patients who died during hospitalization were assigned an NIHSS score of 42 or mRS score of 6. The two outcome endpoints were chosen as BGI at follow-up and 90 days mRS >2.

### 2.3 Imaging protocol and assessment

All patients underwent multimodal stroke-admission CT imaging using a 640-slice multi-detector CT scanner (United Imaging, Shanghai, China). Standard imaging protocols were employed to evaluate the cerebral vascular status. Admission protocols included noncontrast CT, perfusion CT imaging, and CT angiography with the following parameters: tube voltage, 70–90 kV; and tube current, 100–250 mA. A 45 mL iopromide bolus was injected at a flow rate of 4 mL/s, followed by 30 mL saline. The occlusion site was confirmed on CT angiography. The Alberta Stroke Program Early CT Score was assessed using early noncontrast CT. Perfusion CT imaging was used to confirm the core infarction volume using quantitative software. Follow-up noncontrast CT images were obtained using the same scan parameters. Follow-up MRI was performed using a 3.0 T MRI scanner. The MRI sequences included axial T1-weighted, axial T2-weighted, axial fluid-attenuated inversion recovery, and axial diffusion-weighted images with b values of 0 and 1,000 s/mm^2^, as well as automatically calculated apparent diffusion coefficient maps. On follow-up CT or diffusion-weighted images, the BGI final volume was quantified using semi-automated software (uAI workstation). Hemorrhagic transformation was defined as parenchymal hemorrhage or hemorrhagic infarction. Parenchymal hemorrhage was demonstrated as a dense blood clot with a substantial to mild space-occupying effect on the infarction core. Hemorrhagic infarction was defined as sparse hyperdensities within the infarction core or along its margins (21).

### 2.4 Endovascular treatment and angiographic evaluation

**Figure 2:**
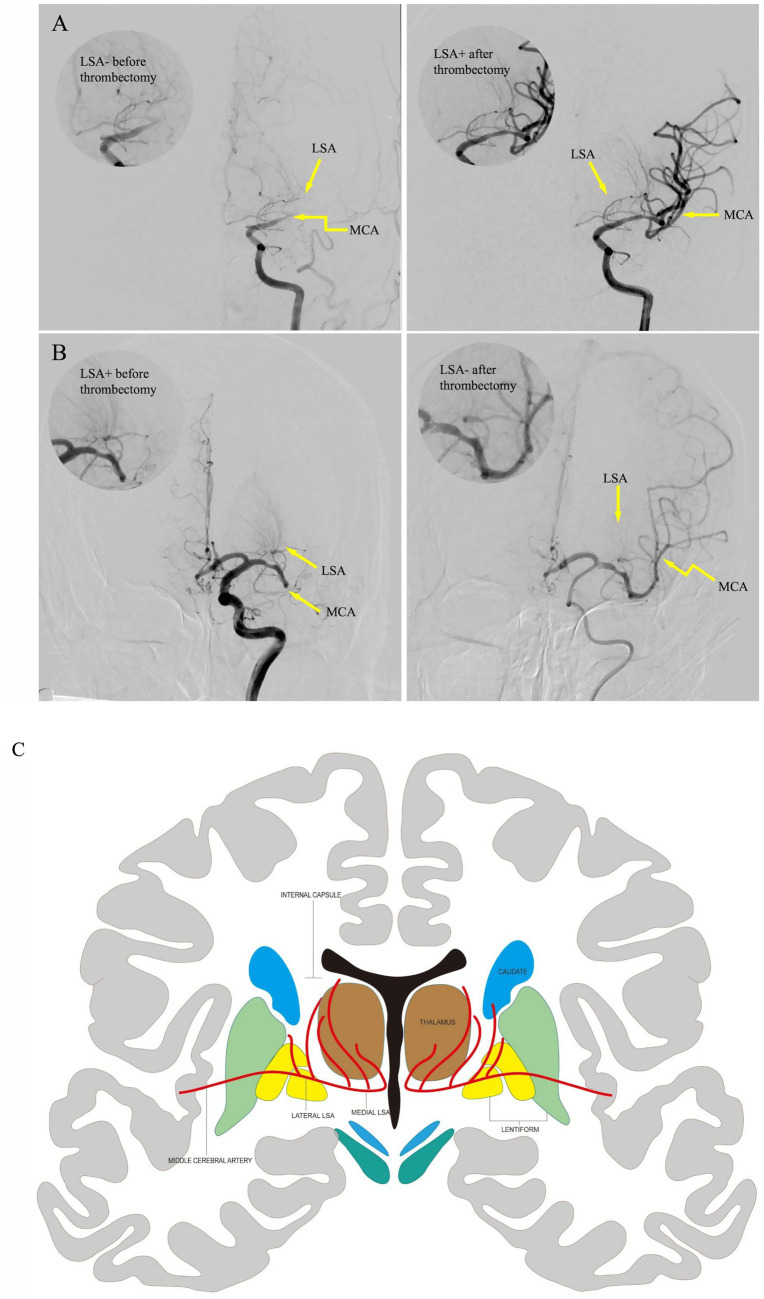
Pre-thrombectomy and post-thrombectomy images of representative LSA recanalization cases. The upper right circle demonstrates LSA perfusion details. Yellow arrows marked the anatomical positions of LSAs and MCA. Case 1 **(A)**. An 80-year-old female patient with proximal M1 occlusion showed faint LSA visualizations on preoperative angiography, which remarkably improved angiographic opacification after mechanical thrombectomy. Case 2 **(B)**. A 59-year-old male patient with distal M1 occlusion exhibited clearly visible LSAs on preoperative angiography, which showed near-complete LSA occlusion with loss of angiographic delineation on post-thrombectomy scan. **(C)** The coronal anatomical illustration of the basal ganglia region, including the MCA perforators, which supplies the nucleus in this region. LSA, lenticulostriate artery; MCA, middle cerebral artery.

Endovascular treatment was performed in the Neuroangiography Suite using a biplane digital angiography machine (Artis Zee; Siemens Healthcare). Patients were treated under conscious sedation or general anesthesia before the procedure. Angiographic features of the LSA were evaluated before and after thrombectomy. The Yaşargil (22) classification was used to classify the LSAs. The morphology of the LSA, including its patency and length, was compared as described in Figures 2A–C; when vessels appeared as continuous dense lines in contrast to adjacent areas, they were classified as LSA+; otherwise, they were classified as LSA−. The pre- and post-thrombectomy LSA sign patterns, from fine to worst, were graded as 1. LSA+/+, 2. LSA−/+, 3. LSA+/−, 4. LSA−/−. LSA+/+ or LSA−/+ were considered favorable LSA patterns. Conversely, LSA+/− and LSA−/− were considered unfavorable patterns. Collateral flow in the ipsilateral ischemic region was assessed based on the American Society of Interventional and Therapeutic Neuroradiology collateral score. Levels 3 and 4 were defined as good collaterals, whereas levels 0–2 were defined as poor. Two board-certified neuroradiologist and neurointerventionalist independently assessed LSA sign patterns and collateral circulation. Discrepancies were resolved through a consensus review. The mTICI grade were assessed at the end of the intervention. The mTICI score of 3 deemed as complete recanalization (23).

### 2.5 Statistical analysis

Continuous variables were described as mean ± standard deviation or median (interquartile range). Student’s *t*-test, Mann–Whitney U test, or Fisher’s exact test was used depending on the data normality. The Kruskal–Wallis H test and chi-square test were used for multi-group comparisons as well as *post hoc* pairwise comparisons. Univariate and multivariate logistic analyses were performed to identify independent parameters after comparing the degrees of collinearity between the two variables. Confounders were selected a priori based on existing literature and clinical consensus (24). Multivariable logistic regression models were used to estimate associations and adjust confounders. Sensitivity analyses were conducted by comparing LSA patterns with adjustment of confounders. All logistic regression results were expressed as odds ratios (OR) or adjusted OR with corresponding 95% confidence intervals. Prediction performance was assessed using receiver operating characteristic (ROC) curve analysis. Goodness of fit was assessed using the Hosmer–Lemeshow test. The area under the curve (AUC) of nested models with and without confounders. Internal validation was performed using 1,000 bootstrap samples. The agreement between observed and predicted outcomes was assessed using calibration plots. Statistical significance was set at two-tailed *p*-values of <0.05. All statistical analyses were performed using SPSS and R software.

## 3 Results

### 3.1 Demographic and clinical characteristics

Initially, 251 participants were enrolled to our cohort. Patients were excluded for posterior circulation occlusion (*n* = 32), missing follow-up imaging (*n* = 29), unsatisfactory recanalization or mTICI less than 2c (*n* = 11) and incomplete medical record (*n* = 21). In total, 158 patients fulfilled the inclusion criteria. The demographic and procedural characteristics of the participants are shown in Table 1. The mean age was 64 ± 14.5 years, and 60.8% (96) participants were male. On admission, hypertension was prevalent in 63.9% (101) of the study cohort. In the study cohort, 25.3% (40) of patients had no BGI and 41.8% (66) achieved good long-term outcome (90-day mRS ≤2). Good long-term outcomes were more prevalent among younger adults and male populations (*p* < 0.001 and *p* = 0.023, respectively). (Supplementary Tables 1, 2). Hypertension, diabetes mellitus, stroke, and atrial fibrillation were associated with poor long-term outcome. The M1 segment was the most frequent occlusion site (54.4%), significantly more common than both M2 and internal carotid artery occlusions (10.1%) in the BGI group (*p* = 0.027). The elevated blood pressure and use of antiplatelets/anticoagulants therapy were more common in BGI and worse long-term outcome. Baseline, discharge NIHSS and discharge mRS scores significantly differ in BGI and 90-day mRS scores. Significantly higher rates of hemorrhagic transformation were observed in patients with BGI (44.9% vs. 15.0%, *p* = 0.001). Additionally, hemorrhagic transformation was significantly associated with both short- and long-term outcomes (*p* = 0.001 and 0.005, Supplementary Figure 1).

**Table 1:** Baseline characteristics of study’s cohort.

Characteristics	Datum
Age (years)	64 ± 14.5
Male	96 (60.8)
Left lesioned side	75 (47.5)
Height (cm)	165 (150, 181)
Weight (kg)	65 (45, 130)
Illness history	
Hypertension	101 (63.9)
Diabetes mellitus	48 (30.4)
Coronary disease	27 (17.1)
Stroke history	38 (24.1)
Atrial fibrillation	43 (27.4)
NIHSS on admission	10 (0,37)
Occlusive vessel site	
M1	106 (67.1)
M2	25 (15.8)
ICA	27 (17.1)
Complete recanalization (mTICI ≥3)	138 (87.3)
Time from angiography to puncture (min)	5 (5, 40)
Time from puncture to recanalization (min)	36 (22, 67)

Data are presented as means ± standard deviations, medians with interquartile ranges in parentheses, or numbers of patients with percentages in parentheses.

ICA, internal carotid artery; mTICI, modified thrombolysis in cerebral infarction.

### 3.2 LSA sign patterns with clinical outcomes

**Figure 3:**
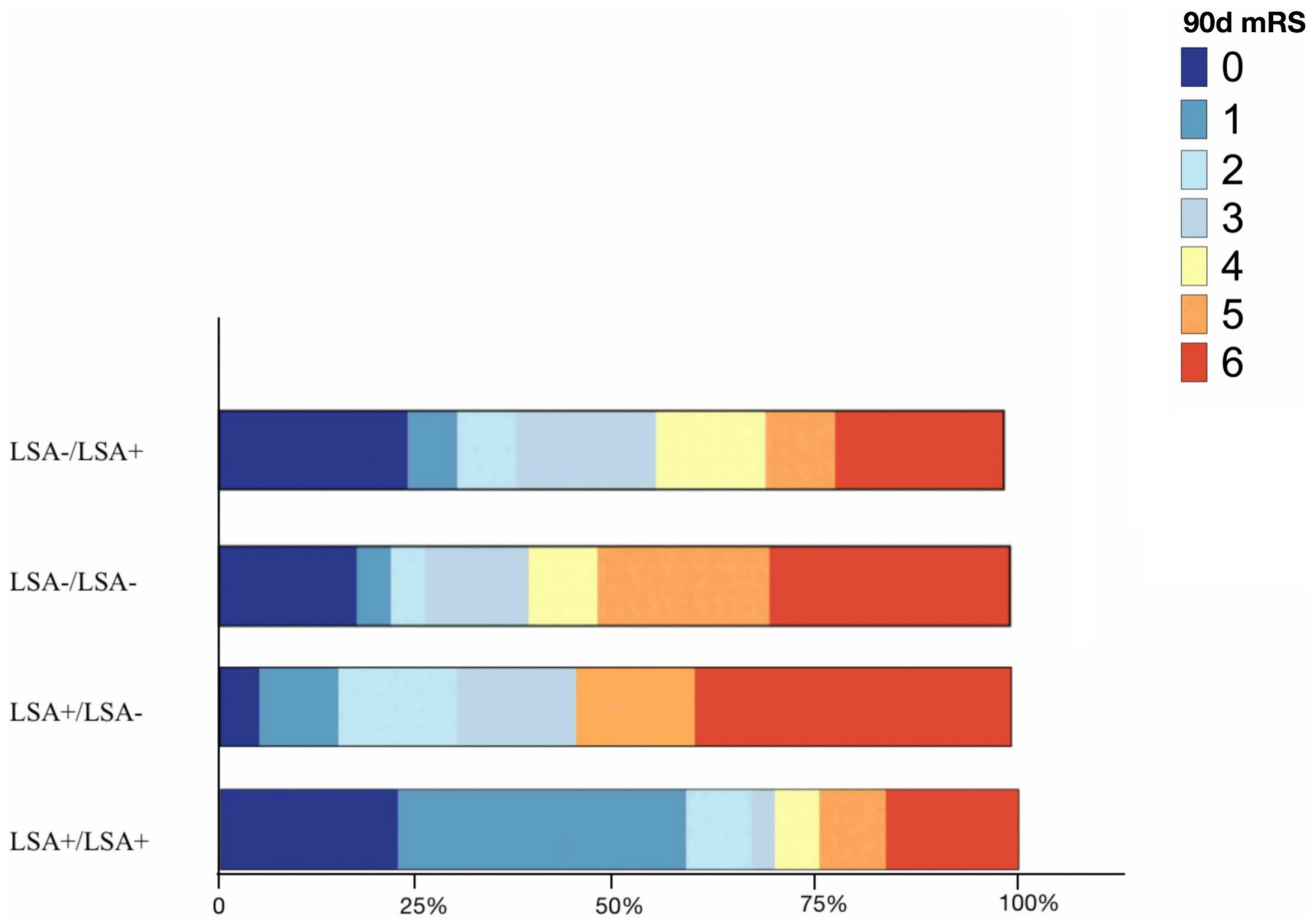
Bar graph depicting the distribution of 90 days mRS in terms of LSA patterns. LSA, lenticulostriate artery; 90 days mRS, 90 days modified Rankin scale.

The LSA sign patterns significantly impacted clinical outcomes in intergroup comparison, as shown in Table 2. Among the patients with patent LSA post-thrombectomy, 36 and 79 were of the LSA+/LSA+ and LSA−/LSA+ groups. The median 90-day mRS scores were 1 and 3, respectively. LSA+ pre- and post-thrombectomy were significantly associated with the absence of BGI after treatment (*p* = 0.026 and 0.023, respectively; Supplementary Table 1). The LSA+/LSA− and LSA+/LSA+ groups demonstrated significantly higher rates of insular lobe infarction than the LSA−/LSA+ group (60 and 63.9% vs. 26.6%, *p* = 0.001). The LSA−/LSA− group showed a greater median infarction volume than the LSA+/LSA+ group (7,732 mm^3^ vs. 620 mm^3^). In intergroup comparison, indistinct LSA post-thrombectomy (LSA−/LSA− and LSA+/LSA−) was associated with more severe long-term outcomes (Figure 3). Good clinical outcomes were observed in the LSA+/LSA+ compared to the LSA+/LSA− group (66.7% vs. 30.0%, *p* = 0.004).

**Table 2:** Intergroup comparison of post-treatment clinical outcomes among different LSA patterns.

Variables	LSA sign patterns				
	LSA−/LSA+ (*n* = 79)	LSA−/LSA− (*n* = 23)	LSA+/LSA− (*n* = 20)	LSA+/LSA+ (*n* = 36)	*p*
Hemorrhage transformation (*n* = 59)	31 (39.2)	11 (47.8)	8 (40.0)	9 (25.0)	0.307
Parenchymal hemorrhage (*n* = 13)	7 (8.9)	3 (13.0)	2 (10.0)	1 (2.8)	
Hemorrhagic infarction (*n* = 46)	24 (30.3)	8 (34.8)	6 (30.0)	8 (22.2)	
Discharge NIHSS	4 (0, 42)	8 (0, 42)	10 (0, 42)	4 (0, 32)	0.094
Discharge mRS	2 (0, 6)	3 (0, 6)	4 (0, 6)	2 (0, 6)	0.204
BGI volume/mm^3^	4,178 (0, 50,310)	7,732 (0, 33,078)a	7,050 (0,28,040)	620 (0, 48,873)	0.004*
BGI evolution					<0.001*
Progression (*n* = 26)	5 (6.3)a	1 (4.3)a	2 (10.0)a	18 (50.0)	
Persistent infarction (*n* = 81)	47 (59.5)a	15 (65.2)a	9 (45.0)	10 (27.8)	
Regression (*n* = 31)	14 (17.7)	5 (21.7)	7 (35.0)	5 (13.9)	
Remained normal (*n* = 20)	13 (16.5)	2 (8.7)	2 (10.0)	3 (8.3)	
Infarction of the LSA territory					
Caudate nucleus (*n* = 43)	16 (20.3)	11 (47.8)	6 (30.0)	10 (27.8)	0.074
Internal capsule (*n* = 52)	25 (31.6)	12 (52.2)	8 (40.0)	7 (19.4)	0.072
Lentiform nucleus (*n* = 114)	60 (75.9)a	20 (87.0)a	16 (80.0)	18 (50.0)	0.006*
Insular lobe (*n* = 65)	21 (26.6)	9 (39.1)	12 (60.0)b	23 (63.9)b	0.001*
Long-term functional outcome (90 days mRS)	3 (0, 6)	5 (0, 6)	5 (0, 6)	1 (0, 6)	0.023*
Good functional outcome (90 days mRS ≤2)	30 (37.9)a	6 (26.1)a	6 (30.0)	24 (66.7)	0.004*

Data are presented as means ± standard deviations, medians with interquartile ranges in parentheses, or numbers of patients with percentages in parentheses. In *post hoc* pairwise comparisons: a, significant compared to LSA+/LSA+ group. b, significant compared to LSA−/LSA+ group.

*p-values indicate the significance of predictors with a threshold of <0.05.

NIHSS, National Institutes of Health Stroke Scale; mRS, modified Rankin scale; LSA, lenticulostriate artery; BGI, basal ganglia infarction; ICA, internal carotid artery.

### 3.3 Associations of LSA patterns for BGI and 90-day mRS score according to the different multivariate models

All variables with statistical significance were included into univariate and multivariate regression analysis (Supplementary Tables 3, 4). Four multivariate regression models were constructed to adjust for potential confounders. In the unadjusted crude model, LSA patterns were positively associated with BGI (*p* = 0.008) as shown in Table 3. In model 1 that adjusted for gender, NIHSS on admission, and occlusive vessel site, LSA patterns remained positively associated with BGI (*p* = 0.030). In model 2, after additional adjustment of peri-procedural variables, LSA patterns remained positively associated with BGI (*p* = 0.037). In Table 4, model 3 adjusted for age, gender, hypertension, diabetes mellitus, stroke history, atrial fibrillation, and NIHSS on admission. Model 4 adjusted for peri-procedural variables. After adjusting all confounders, LSA sign patterns and discharge mRS score remained independent predictors for 90 day mRS >2 (*p* = 0.001 and 0.008).

**Table 3:** Association of LSA patterns with BGI.

Primary outcome: BGI	Crude Model 1	Adjusted Model 1			Adjusted Model 2	
Variables	Unadjusted OR	*p*	Adjusted OR	*p*	Adjusted OR	*p*
LSA patterns		0.008*		0.030*		0.037*
LSA−/LSA+	1		1		1	
LSA−/LSA−	3.88 (0.55, 45.77)		1.96 (0.41, 14.72)		2.95 (0.72, 20.16)	
LSA+/LSA−	1.07 (0.21, 6.06)		1.02 (0.23, 4.77)		0.97 (0.28, 3.92)	
LSA+/LSA+	0.17 (0.04, 0.61)		0.34 (0.11, 0.88)		0.39 (0.16, 0.94)	
Hemorrhagic transformation	5.29 (1.64, 20.27)	0.008*	4.25 (1.49, 14.12)	0.012*	3.62 (1.22, 10.48)	0.009*
Discharge mRS	0.97 (0.71, 1.31)	0.852	1.07 (0.85, 1.36)	0.563	1.19 (0.98, 1.46)	0.081

Crude model 1 adjusted for none.

Adjusted Model 1: adjusted for gender, NIHSS score on admission, and occlusive vessel site.

Adjusted Model 2: adjusted for SBP and DBP in addition to the variables in Adjusted Model 1.

Data are presented as odds ratio with 95% confidence interval in parentheses.

*p-values indicate the significance of predictors with a threshold of <0.05.

BGI, basal ganglia infarction; NIHSS, National Institutes of Health Stroke Scale; mRS, modified Rankin Scale; LSA, lenticulostriate artery; OR, odds ratio.

**Table 4:** Association of LSA patterns with long-term outcome.

Secondary outcome: 90 days mRS >2	Crude Model 2		Adjusted Model 3		Adjusted Model 4	
Variables	Unadjusted OR	*p*	Adjusted OR	*p*	Adjusted OR	*p*
LSA patterns		0.002*		0.002*		0.001*
LSA−/LSA+	1		1		1	
LSA−/LSA−	1.98 (0.39, 11.64)		2.61 (0.58, 13.42)		1.68 (0.42, 7.02)	
LSA+/LSA−	0.31 (0.03, 3.81)		0.34 (0.06, 1.78)		0.39 (0.08, 1.80)	
LSA+/LSA+	0.07 (0.01, 0.35)		0.09 (0.02, 0.39)		0.10 (0.02, 0.36)	
Hemorrhagic transformation	1.79 (0.51, 6.74)	0.372	1.20 (0.42, 3.39)	0.743	1.52 (0.58, 4.05)	0.382
Discharge NIHSS	1.14 (1.01, 1.34)	0.063	1.13 (1.01, 1.30)	0.052	1.18 (1.05, 1.35)	0.010*
Discharge mRS	1.81 (1.05, 3.24)	0.032*	1.91 (1.25, 3.01)	0.003*	1.70 (1.14, 2.53)	0.008*

Crude Model 2 adjusted for none.

Adjusted Model 3: adjusted for age, gender, hypertension, diabetes mellitus, stroke history, atrial fibrillation, and NIHSS score on admission.

Adjusted Model 4: adjusted for SBP and antiplatelet/anticoagulant therapy in addition to the variables in Adjusted Model 3.

Data are presented as odds ratio with 95% confidence interval in parentheses.

*p-values indicate the significance of predictors with a threshold of <0.05.

NIHSS, National Institutes of Health Stroke Scale; mRS, modified Rankin Scale; LSA, lenticulostriate artery; OR, odds ratio.

### 3.4 ROC analysis

**Figure 4:**
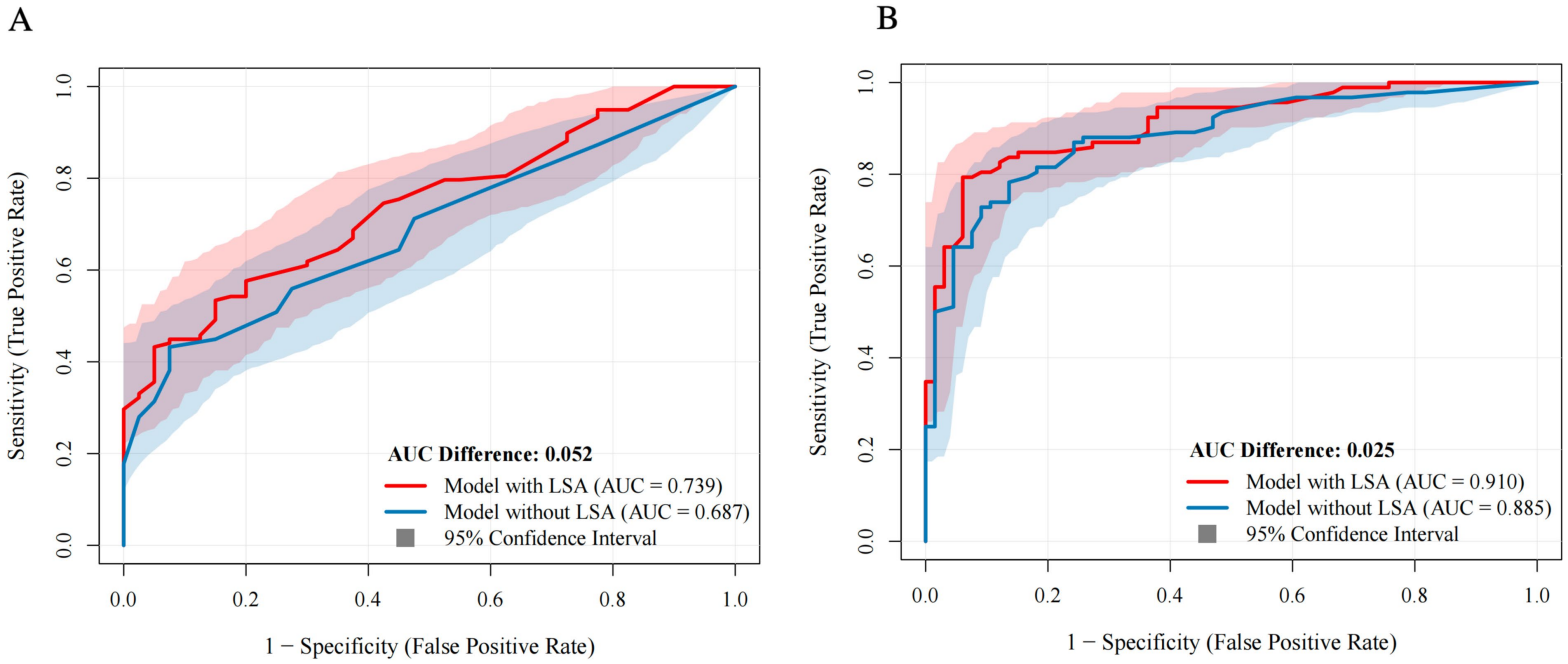
Comparison of prediction models with versus without LSA combination. Blue curves represent models without LSA patterns. Red curves represent models with LSA patterns [**(A)** BGI; **(B)** Long-term outcome]. Shaded area indicates confidence intervals. LSA, lenticulostriate artery; AUC, area under the receiver operating characteristic curve.

The AUC the nested models for BGI ranged between 0.74–0.92, with or without adjustment for confounders (Supplementary Table 5). Regarding long-term outcome prediction, the AUC of the predictive models ranged between 0.91–0.96. The ROC curves of the prediction models combining LSA patterns were compared with prediction models without LSA patterns, shown in Figure 4. Prediction models without LSA patterns had lower AUC than models combining LSA patterns (AUC: 0.74 vs. 0.69 and 0.91 vs. 0.89). The bootstrap C-index values of the prediction models for BGI and 90-day mRS score were 0.78 and 0.97, respectively. Both prediction models exhibited better calibration and discrimination ability (Hosmer–Lemeshow test *χ*^2^ = 4.59, *p* = 0.830).

## 4 Discussion

To the best of our knowledge, this is the first study to characterize pre- and post-thrombectomy LSA angiographic signs in patients with large-vessel occlusion. Our study suggests that LSA sign patterns are significantly associated with BGI and long-term functional independence. Our models comprising clinical and imaging markers demonstrated good prediction performance for both BGI and 90-day mRS scores (AUCs, 0.74 and 0.91, respectively) and may thereby play a promising role in facilitating treatment strategies following thrombectomy.

While a number of studies have explored the prognostic value of LSA visualization in stroke triage. Kaesmacher et al. (12) reported that the appearance of LSAs on magnetic resonance angiography after thrombectomy was associated with favorable outcomes. Horie et al. (25) suggested that the basal ganglia fate was associated with the involvement of LSA. In contrast, other studies have shown that pre-thrombectomy LSA visualization or MCA occlusion involving LSA also correlates with prognosis (11, 16, 26). In the current study, we characterize the LSA dynamic change during thrombectomy, integrating both pre- and post-treatment imaging to identify patterns. Notably, this method relies on standard DSA during the intervention course, avoiding additional appointment registration or scheduling delays. Despite its clinical convenience, the utility of the dynamic LSA patterns for prediction purpose remained underexplored. Our findings address this gap by using LSA status as independent predictor for both BGI and 90-day functional outcomes, even after adjusting for multiple clinical and radiological confounders. After adjustment risk factor of age, gender, medical history, admission NIHSS, occlusion site and peri-procedural factors, the associations between LSA patterns and predicting BGI as well as long-term mRS outcomes remained statistically significant, confirming its independent prognostic value.

Our intergroup comparison found that imaging and clinical outcomes varied widely based on LSA sign patterns, with favorable post-thrombectomy LSA patterns associated with a good long-term prognosis. For instance, patients with patent LSA throughout the intervention (LSA+/LSA+ group) demonstrated a median 90-day mRS score of 1, which is lower than the median 90-day mRS score of those with unfavorable LSA patterns after thrombectomy (LSA−/LSA− and LSA+/LSA− groups). Regarding the unfavorable LSA patterns before and after treatment, the lentiform nucleus was found to be most vulnerable to ischemia, followed by the insular lobe, caudate nucleus, and internal capsule. As previously described, this is due to the differences in the cellular constituents and metabolic demands of the regional territories. The caudate nucleus, lentiform nucleus, and insular lobes, which are composed of gray matter, are relatively more sensitive to anoxic environments. Conversely, the internal capsule, which is composed of white matter (27), is less vulnerable to ischemia after thrombectomy (28, 29).

The phenomenon of unfavorable LSA vascular patterns during thrombectomy result in poor prognosis could be interpreted. Certain neuroimaging markers of LSA morphology have been proposed to reflect vascular vulnerability (12, 29, 30). Because of the congenital absence of anastomotic branches of perforating arteries, this vascular anatomy is inherently predisposed to lenticulostrate infarction depending on occlusion site of perforating arteries (31). In addition, microvascular hemodynamic changes may contribute to this vulnerability. Studies have shown that lack of dilated or preserved LSAs on follow-up MRA may associated with less favorable functional outcomes, possibly impacting microvascular integrity and more extensive reperfusion injury (12, 30). Prolonged hypoperfusion or delayed reperfusion following large-vessel occlusion damage the cerebral autoregulatory mechanisms in small perforating arteries, lead to infarction in ischemia-prone territories such as the striatocapsular region. Other factors such as inflammatory responses and thrombus migration have also been implicated as mechanisms that may impair vascular response to ischemia (32, 33).

Moreover, we found that hemorrhagic transformation was not associated with LSA patency. However, its incidence was higher with BGI. After spontaneous reperfusion, a series of changes including activation of the endothelium and excessive production of oxygen-free radicals can compromise the integrity of the blood–brain barrier within infarcted areas (34). Despite successful recanalization, hyperperfusion and hemodynamic changes in the infarcted tissue may occur. These hemodynamic changes, especially in the LSA territory where leptomeningeal collaterals are scarce, are associated with a higher risk of hemorrhagic transformation (35). Nevertheless, the neurological status at discharge could have more impact long-term outcomes than BGI and hemorrhagic transformation. BGI is more closely associated with cognitive impairment (10). The incorporation of discharge NIHSS and discharge mRS scores into the second model aligns with previous research (10, 25). The early neurological exam could predict long-term neurological function status and the daily activities rehabilitation.

Importantly, our finding regarding LSA signs was consistent with previous studies, supporting the use of LSA patterns for predicting outcomes in the striatocapsular region and prognostication after endovascular therapy (12, 26). Moreover, its predictive accuracy was on par with existing models that rely on angiography imaging for prognosis (36). Our findings are consistent with recent studies, and our investigation of vascular imaging markers further supports current systematic review evidence. Our investigation of vascular imaging markers further supports current systematic review evidence (17). This underscore the pragmatical use of intra-procedural angiographic signs, such as LSA patterns, to guide prognosis without the need for costly additional imaging or waiting for long-term evaluation.

This study has several limitations. First, this was a retrospective study with a relatively small sample size. Large-sample prospective studies are warranted for external validation of the clinical applicability of the proposed predictive model. Second, heterogeneity in the clinical background and management of the patients, including discharge medication, was not controlled and may have influenced the prognosis and, thus, the performance of our model. Finally, a longer follow-up period should be considered to assure more accurate evaluation of the long-term predictive performance of the model.

In conclusion, our findings underscore the importance of routine monitoring of the LSA sign in patients with large-vessel occlusion undergoing thrombectomy. Our proposed models demonstrated good predictive performance for both BGI and 90-day mRS scores following mechanical thrombectomy. Further clinical studies are warranted to validate our findings and explore the role of LSA sign patterns in predicting interventional surgical outcomes in large-vessel occlusions.

## Data Availability

The raw data supporting the conclusions of this article will be made available by the authors, without undue reservation.
