# Supplementary material for: Angiographic assessment of lenticulostriate artery sign to predict clinical outcomes after thrombectomy in patients with stroke
Source: Front Neurol. 2025 Aug 15;16:1644288. doi: 10.3389/fneur.2025.1644288 (PMC12394054; doi:10.3389/fneur.2025.1644288)
Supplement: Supplementary file 1 [file Data_Sheet_1.PDF]

Supplementary Table 1. Summary of baseline, procedural, imaging characteristics between BGI and without BGI.

	Without BGI(n=40)	BGI(n=118)	P
Age	68±2.1	70±1.4	0.292
Gender(Male)	32(80.0)	64(54.2)	0.004*
Side(Left)	17(42.5)	58(49.2)	0.464
Illness history			
Hypertension	27(67.5)	74(62.7)	0.593
Diabetes mellitus	17(42.5)	31(26.3)	0.054
Coronary disease	8(20.0)	19(16.1)	0.576
Stroke history	11(27.5)	27(22.9)	0.562
Atrial fibrillation	12(30.0)	31(26.5)	0.684
NIHSS on admission	9(1,37)	12(0,35)	0.036*
ASPECTS	8(6,10)	8(4,10)	0.673
Occlusive vessel site			0.027*
M1	20(12.7)	86(54.4)	
M2	9(5.7)	16(10.1)	
ICA	11(7.0)	16(10.1)	
Peri-procedural care			
SBP (mmHg)	100(94, 104)	123(105,135)	0.001*
DBP (mmHg)	71(64, 78)	87(78, 96)	0.020*
Antiplatelets/anticoagulants therapy	19(47.5)	58(49.2)	0.029*
Infection status: aberrant	3(7.5)	16(13.6)	0.304
Coagulation status: aberrant	18(45.0)	35(29.7)	0.072
C-reactive protein, mg/L	4.6(1.2,17.5)	14(9.5,76.1)	0.296
LSA+ before thrombectomy	20(50.0)	36(30.5)	0.026*
LSA+ after thrombectomy	35(87.5)	81(69.2)	0.023*
Good collateral circulation	32(80.0)	86(72.9)	0.374
Hemorrhagic transformation	6(15.0)	53(44.9)	0.001*
Discharge NIHSS	2(0,42)	4(0,42)	0.062
Discharge mRS	2(0, 6)	3(0, 6)	0.014*

Note: BGI, basal ganglia infarction. NIHSS, National Institutes of Health Stroke Scale;ASPECTS, the Alberta Stroke Programme Early CT Score;ICA, internal carotid artery;SBP, systolic blood pressure; DBP, diastolic blood pressure; LSA, lenticulostriate artery; mRS, modified Rankin Scale.

Supplementary Table 2. Summary of baseline, procedural, imaging characteristics between 90d mRS≤2 and > 2.

	90d mRS≤2(n=66)	90d mRS > 2(n=92)	P
Age	64±2.7	73±1.5	<0.001*
Gender(Male)	47(71.2)	49(53.3)	0.023*
Side(Left)	40(60.6)	48(52.2)	0.347
Illness history			
Hypertension	32(48.5)	69(75.0)	0.001*
Diabetes mellitus	13(19.7)	35(38.0)	0.013*
Coronary disease	9(13.6)	18(19.6)	0.336
Stroke history	8(12.1)	30(32.6)	0.003*
Atrial fibrillation	9(13.8)	34(37.0)	0.010*
NIHSS on admission	8(0,29)	14(2,35)	<0.001*
ASPECTS	6(4,10)	8(3,10)	0.632
Occlusive vessel site			0.063
M1	51(32.3)	55(34.8)	
M2	8(5.1)	17(10.8)	
ICA	7(4.4)	20(12.7)	
Peri-procedural care			
SBP (mmHg)	105(96, 113)	132(116, 152)	0.001*
DBP (mmHg)	82(71,92)	86(71, 96)	0.092
Antiplatelets/anticoagulants therapy	23(34.8)	54(58.7)	0.030*
Infection status: aberrant	4(6.1)	15(16.3)	0.532
Coagulation status: aberrant	20(30.3)	33(35.9)	0.475
C-reactive protein, mg/L	4.9(1.0, 19.4)	12.4(10.2, 65.3)	0.357
LSA+ before thrombectomy	30(45.4)	26(28.3)	0.026*
LSA+ after thrombectomy	54(81.8)	62(68.1)	0.054
Region of the BGI			0.742
Caudate nucleus	19(28.8)	24(26.1)	0.715
Internal capsule	18(27.3)	32(34.8)	0.321
Lentiform nucleus	44(66.7)	70(76.1)	0.227
Insular lobe	28(42.4)	37(40.2)	0.781
Good collateral circulation	52(78.8)	66(71.7)	0.175
Hemorrhagic transformation	16(24.2)	43(46.7)	0.005*
Discharge NIHSS	1(0,12)	8(0,42)	<0.001*
Discharge mRS	1(0,4)	4(1,6)	<0.001*

Note: BGI, basal ganglia infarction. NIHSS, National Institutes of Health Stroke Scale;ASPECTS, the Alberta Stroke Programme Early CT Score;ICA, internal carotid artery;SBP, systolic blood pressure; DBP, diastolic blood pressure; LSA, lenticulostriate artery; mRS, modified Rankin Scale.

Supplementary Table 3. Univariate analysis of baseline, procedural, imaging variables with BGI and 90d mRS.

	BGI		90d mRS>2	
Variables	OR	P	OR	P
Age	-	-	1.06(1.03 1.08)	<0.001*
Gender(Male)	3.38(1.43, 7.93)	0.005 *	2.17(1.12, 4.31)	0.022*
NIHSS on admission	1.04(0.99,1.09)	0.075	1.08(1.04,1.13)	0.001*
Hypertension	-	-	3.18(1.63, 6.33)	<0.001*
Diabetes mellitus	-	-	2.50(1.22, 5.38)	0.014*
Stroke history	-	-	3.51(1.55, 8.78)	0.004*
Atrial fibrillation	-	-	3.64(1.66, 8.72)	0.002*
Occlusive vessel site		0.031*	-	-
M1	1		-	-
M2	0.41(0.16, 1.07)		-	-
ICA	0.34(0.14, 0.84)		-	-
SBP(mmHg)	1.02(1.01, 1.05)	0.002*	1.05(1.03, 1.07)	<0.001*
DBP (mmHg)	1.09(1.05, 1.13)	0.001*	-	-
Antiplatelets/anticoagulants therapy	1.06(0.52, 2.20)	0.853	2.65(1.39, 5.17)	0.003*
LSA patterns		0.020*		0.007*
LSA-/LSA+	1		1	
LSA-/LSA-	3.10(0.66, 14.50)		1.73(0.62, 4.89)	
LSA+/LSA-	1.18(0.35, 3.98)		1.43(0.50, 4.12)	
LSA+/LSA+	0.37(0.16, 0.86)		0.31(0.13, 0.70)	
Hemorrhagic transformation	1.56(0.84, 2.21)	0.001*	4.62(1.80, 11.84)	0.001*
Discharge NIHSS	-	-	1.27(1.16, 1.40)	0.001 *
Discharge mRS	1.25(1.04, 1.51)	0.017*	2.29(1.79, 2.94)	0.001 *

Note: BGI, basal ganglia infarction; mRS, modified Rankin Scale; OR, odds ratio; NIHSS, National Institutes of Health Stroke Scale; ICA, internal carotid artery; SBP, systolic blood pressure; DBP, diastolic blood pressure; LSA, lenticulostriate artery.

Supplementary Table 4. Multivariate analysis of baseline, procedural, imaging variables with BGI and 90d mRS.

	BGI		90d mRS>2	
Variables	OR	P	OR	P
Age	-	-	1.02(0.98, 1.07)	0.325
Gender(Male)	5.39 (1.60, 21.91)	0.012*	1.68 (0.43, 7.00)	0.465
NIHSS on admission	1.04 (0.98, 1.12)	0.167	0.97 (0.89, 1.04)	0.424
Hypertension	-	-	2.06(0.52, 4.42)	0.306
Diabetes mellitus	-	-	3.78 (0.91, 17.36)	0.073
Stroke history	-	-	3.47 (0.64, 24.07)	0.166
Atrial fibrillation	-	-	3.43 (0.67, 20.26)	0.151
Occlusive vessel site		0.006*	-	-

M1	1		-	-
M2	0.11 (0.02, 0.49)		-	-
ICA	0.06 (0.01, 0.28)		-	-
SBP(mmHg)	1.02 (0.98, 1.05)	0.091	1.06 (1.03, 1.10)	2.432
DBP (mmHg)	1.10 (1.06, 1.16)	0.001*	-	-
Antiplatelets/anticoagulants therapy	-	-	1.31 (0.32, 5.06)	0.694
LSA patterns		0.008*		0.002*
LSA-/LSA+	1		1	
LSA-/LSA-	3.88 (0.55, 45.77)		1.98(0.39, 11.64)	
LSA+/LSA-	1.07 (0.21, 6.06)		0.31 (0.03, 3.86)	
LSA+/LSA+	0.17 (0.04, 0.61)		0.07(0.01, 0.35)	
Hemorrhagic transformation	5.29 (1.64, 20.27)	0.008*	1.79 (0.51, 6.74)	0.371
Discharge NIHSS	-	-	1.14 (1.01, 1.34)	0.063
Discharge mRS	0.97(0.71, 1.31)	0.855	1.81(1.05, 3.24)	0.032*

Note: BGI, basal ganglia infarction; mRS, modified Rankin Scale; OR, odds ratio; NIHSS, National Institutes of Health Stroke Scale; ICA, internal carotid artery; SBP, systolic blood pressure; DBP, diastolic blood pressure; LSA, lenticulostriate artery.

Supplementary Table 5. The receiver operating characteristic curve analysis of all prediction models.

	AUC	Sen	Spe	Acc	Cut-off
Crude Model 1	0.92(0.87,0.96)	0.86	0.85	0.85	0.72
Adjusted Model 1	0.85(0.77,0.93)	0.84	0.78	0.82	0.62
Adjusted Model 2	0.74(0.66,0.82)	0.53	0.85	0.62	0.82
Crude Model 2	0.96(0.93,0.98)	0.86	0.95	0.90	0.69
Adjusted Model 3	0.93(0.90,0.97)	0.86	0.88	0.87	0.59
Adjusted Model 4	0.91(0.87,0.96)	0.79	0.94	0.85	0.66

Crude model 1 and 2 adjusted for: None.

Adjusted Model 1: Adjusted for gender, NIHSS score on admission, and occlusive vessel site.

Adjusted Model 2: Adjusted for SBP and DBP in addition to the variables in Adjusted Model 1.

Adjusted Model 3: Adjusted for age, gender, hypertension, diabetes mellitus, stroke history, atrial fibrillation, and NIHSS score on admission.

Adjusted Model 4: Adjusted for SBP and antiplatelet/anticoagulant therapy in addition to the variables in Adjusted Model 3.

Note: AUC, area under the receiver operating characteristic curve; Sen, sensitivity; Spe, specificity; Acc, accuracy.

Supplementary Figure 1. Percentage of hemorrhage transformation in association of different BGI and long-term outcome.

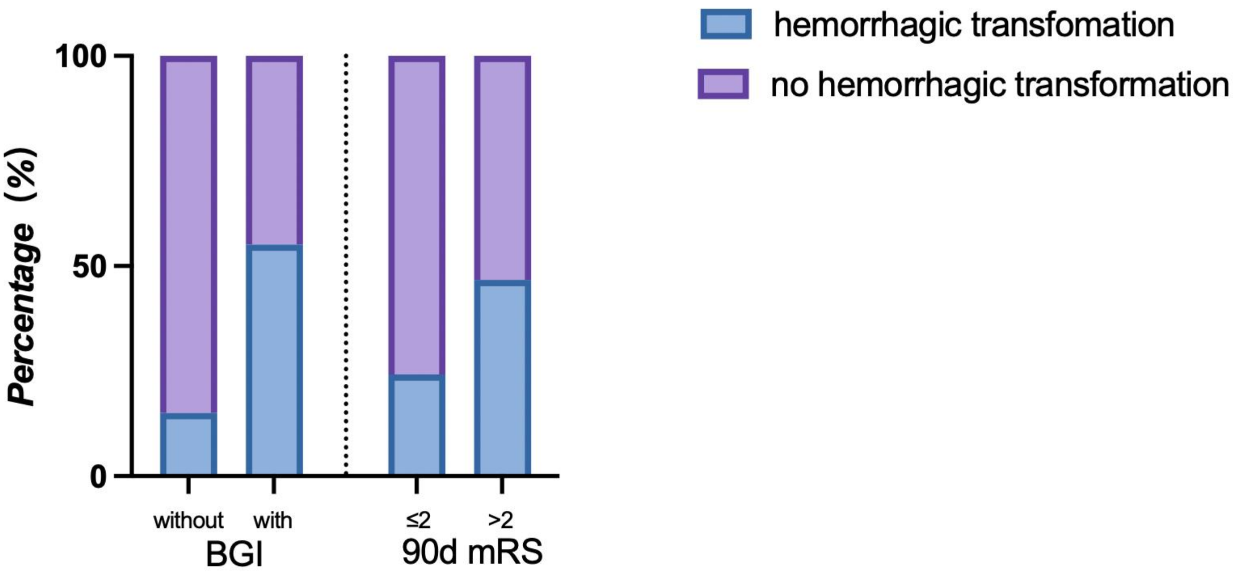

Note: BGI, basal ganglia infarction; mRS, modified Rankin Scale.

Supplementary Figure 2. Calibration plots illustrating the bootstrap-validated performance of the prediction model for (A) BGI and (B) 90d mRS>2.

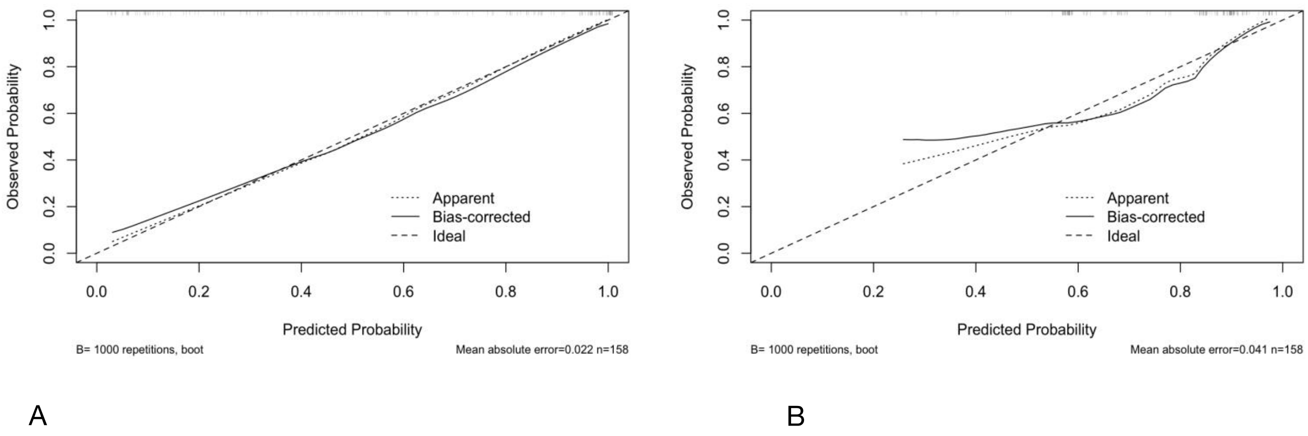

Note: BGI, basal ganglia infarction; mRS, modified Rankin Scale.
